# Anti‐Wound Dehiscence and Antibacterial Dressing with Highly Efficient Self‐Healing Feature for Guided Bone Regeneration Wound Closure

**DOI:** 10.1002/adhm.202304128

**Published:** 2024-03-08

**Authors:** Shenghao Xue, Ning Tang, Cheng Zhou, Shuobo Fang, Hossam Haick, Jiao Sun, Xueying Wu

**Affiliations:** ^1^ Department of Prothodontics Shanghai Stomatological Hospital & School of Stomatology Shanghai Key Laboratory of Craniomaxillofacial Development and Diseases Fudan University Shanghai 200001 P. R. China; ^2^ Precision Research Center for Refractory Diseases in Shanghai General Hospital Shanghai Jiao Tong University School of Medicine Shanghai 200025 China; ^3^ School of Electronic Information and Electrical Engineering Shanghai Jiao Tong University Shanghai 200240 P. R. China; ^4^ Department of Chemical Engineering and Russell Berrie Nanotechnology Institute Technion‐Israel Institute of Technology Haifa 3200003 Israel; ^5^ Department of Dental Materials Shanghai NinthPeople's Hospital Shanghai Jiao Tong University School of Medicine National Clinical Research Center for Oral Diseases Shanghai Key Laboratory of Stomatology Shanghai 200011 P. R. China

**Keywords:** antibacterial, bone regeneration, dressing, self‐healing, wound closure

## Abstract

Guided bone regeneration (GBR) is a well‐established technique for preserving and enhancing alveolar ridge structures. Success in GBR relies on fulfilling the Primary wound closure, Angiogenesis, Space maintenance, and Stability (PASS) principles. Conventional methods, involving titanium meshes and sutures, have drawbacks, including the need for secondary removal and customization challenges. To address these issues, an innovative multifunctional GBR dressing (MGD) based on self‐healing elastomer (PUIDS) is introduced. MGD provides sutureless wound closure, prevents food particle accumulation, and maintains a stable environment for bone growth. It offers biocompatibility, bactericidal properties, and effectiveness in an oral GBR model. In summary, MGD provides a reliable, stable osteogenic environment for GBR, aligning with PASS principles and promoting superior post‐surgery bone regeneration.

## Graphical abstract

The novel multifunctional dressing (MGD) for oral guided bone regeneration (GBR) features biocompatibility, antibacterial properties, self‐healing, and water repellence, preventing wound dehiscence and contamination. It adheres to “PASS” principles, supports graft material, and promotes early bone formation, revolutionizing oral GBR management.

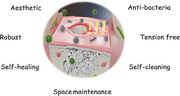

## 1 Introduction

Guided bone regeneration (GBR), first described by Dahlin in 1988,^[^ ^1^ ^]^ is one of the most common strategies for alveolar ridge preservation/augmentation and regarded as a standard treatment modality.^[^ ^2^, ^3^ ^]^ To ensure the success of GBR surgery, predictable bone regeneration requires four major biologic principles, including primary wound closure, angiogenesis, space maintenance, and stability (PASS).^[^ ^4^, ^5^ ^]^ When the PASS principle cannot be well met, it is easy to produce soft tissue complications, such as membrane exposure, soft tissue dehiscence, and acute infection or abscess, with the prevalence of 16.8%,^[^ ^6^, ^7^ ^]^ leading to the impaired wound healing and insufficient bone regeneration. Therefore, how to achieve wound closure on the basis of effectively ensuring the reliable stability of bone graft materials in space and time to meet the PASS principle is a prerequisite to provide guarantee for the GBR effect.

Conventional approaches such as the use of titanium meshes and pins for membrane fixation have been employed to ensure the space maintenance and stability of bone grafts.^[^ ^8^, ^9^ ^]^ However, these metal accessories must be removed in an additional surgical procedure, resulting in secondary trauma.^[^ ^10^ ^]^ In addition, shaping titanium mesh can often be a highly challenging task that frequently requires personalized customization, which in turn incurs additional time and expenses.^[^ ^11^ ^]^ On the other hand, in clinical practice, soft tissue wound closure is typically achieved using sutures or absorbable threads. Unfortunately, this can lead to the accumulation of food particles, which in turn can easily cause bacteria multiply, increasing the risk of infection.^[^ ^12^ ^]^ Additionally, the increased tension in soft tissues following bone grafting can frequently lead to tissue splitting.^[^ ^13^, ^14^ ^]^ Due to the aforementioned issues, localizing the bone grafting material in clinical practice becomes challenging, particularly in the case of larger bone defects, making it difficult to achieve spatial and temporal stability.^[^ ^15^ ^]^

Currently, to attain optimal healing and adhere to the “PASS” principles, a series of innovative methods and materials have been widely studied. The photocrosslinked collagen membrane can provide spatio‐temporal support to a certain extent and prevent the leakage of bone graft materials.^[^ ^16^ ^]^ However, due to their location beneath the incision, these modified membranes are unable to achieve tension‐free primary wound closure while maintaining the incision space. Multiple adhesive and self‐healing hydrogels can improve the binding strength of collagen membrane in wet bone surfaces,^[^ ^17^, ^18^ ^]^ and also have the ability to assist wound closure. Moreover, the rich bioactive substances contained within them can promote wound healing,^[^ ^19^ ^]^ control inflammation,^[^ ^20^ ^]^ and inhibit antimicrobial activity.^[^ ^21^ ^]^ Nevertheless, their utilization in the moist oral environment is limited, and their mechanical strength is often insufficient to maintain adequate space for bone growth. Therefore, there is still an urgent need for a new postoperative soft tissue management method that can provide a reliable and stable osteogenic space for the GBR process on the basis of effectively achieving wound closure.

Considering the above‐mentioned limitations, we have introduced an innovative multifunctional GBR dressing (MGD) based on self‐healing elastomer (PUIDS). The MGD was synthesized by one‐pot method and had favorable mechanical toughness and self‐healing ability, which can not only achieve sutureless wound closure, but also effectively maintains the bone growth space, playing a crucial role in the subsequent osteogenesis process. Related experiments exhibited that the MGD had desirable biocompatibility and bactericidal ability, and could prevent the accumulation of food residues at the wound site. Moreover, a rabbit oral GBR model was constructed, instead of using cranial or tibial bones, to evaluate the performance of MGD in practical applications. According to the surface and structural results in animal models, MGD could assist wound closure and effectively avoid the loss of bone graft materials. As proof‐of‐concept of a novel GBR dressing, the management strategy presented here can provide durable and reliable protection for the GBR process while adhering to the “PASS” principle.

## 2 Results and Discussion

### 2.1 Preparation and Characterization of Self‐Healing Elastomer

**Figure 1:**
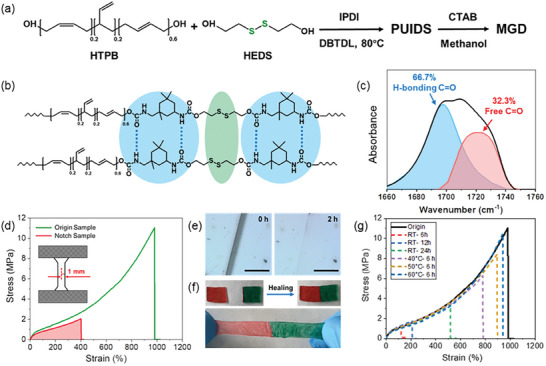
General design and characterizes of MGD. a) Synthetic procedure for its preparation. b) Proposed ideal structure and self‐healing mechanism of the MGD network based on reversible disulfide and hydrogen bonds. c) The stretching vibration region of free C = O groups as well as H‐bonded C = O groups at 1727 cm‐1 and 1697 cm‐1, respectively. d) Typical stress‐strain curves of original and notched MGD samples (gauge length: 16 mm; width: 2 mm; thickness: 0.45–0.55 mm). e) Images of the scratched MGD in the self‐healing process at 60 °C, scale bar: 100 µm. f) Photographs of MGD after healing for 30 min at room temperature (top) and stretched (bottom). g) Stress‐strain curves of original and full‐cut MGD samples healed at different times at room temperature and at 40–60 °C for 6 h.

The preparation of the self‐healing polymer with high mechanical strength and antibacterial properties through a facile strategy is illustrated in **Figure** **1**a. First, one‐pot polycondensation reaction was used to synthesis the prepolymer (PUIDS, Figure S1, Supporting Information). The structural information of PUIDS was depicted in Figure S2 (Supporting Information). Thereafter, PUIDS was re‐dissolved with chloroform, and a certain amount of cetyltrimethylammonium bromide (CTAB) was added to form a uniform mixture. Pouring the mixture into the mold to make the solvent evaporate, the final elastomer (MGD) can be obtained. The HEDS and isophorone diisocyanate (IPDI) together contribute dynamic covalent (disulfide) and noncovalent bonds (hydrogen bonds), which is very important for the efficient self‐healing of the elastomer (Figure 1b). To calculated the degree of microphase separation (DPS), the stretching vibration region of free C = O groups (1727 cm^−1^) and H‐bonded C = O groups (1697 cm^−1^) was fitted using Gauss‐Lorenz curves.^[^ ^22^, ^23^ ^]^ A 67.7% of DPS demonstrated that the MGD has a high degree of hydrogen bonding, which induces a strong phase separation of hard and soft segments, resulting in an improved tear resistance and toughness of MGD (Figure 1c). Furthermore, MGD exhibited good stability below 240 °C (Figure S3, Supporting Information). The average transmittance of MGD film with a thickness of 200 µm under visible light wavelengths was >97% (Figure S4, Supporting Information), which could effectively ensure oral aesthetics during GBR surgery.

To obtain effective wound closure and fixation of bone meal after GBR surgery, the elastomer should have excellent mechanical strength and self‐healing abilities. In terms of MGD, the hydrogen and disulfide bonds break and recombine as crosslinking points during stretch‐recovery process (Figure S5, Supporting Information),^[^ ^24^, ^25^ ^]^ giving MGD an ultimate tensile strength of 11.04 MPa and a tensile strain of ≈980%, with an outstanding toughness of 41.22 MJ m^−3^ (Figure 1d). Excellent tear resistance is key to avoid the effects of soft tissue tension, for which we performed tensile tests in MGD pre‐damaged samples (notched size is half the width of pristine specimen). From the results we could notice that the notch passivation during the tensile process, and could withstand up to 400% strain (Figure 1d; Figure S6 in Supporting Information). The calculated fracture energy is 3.1 kJ m^−2^ using the Greensmith method, indicating that the elastomer has a favorable notch insensitivity.^[^ ^26^ ^]^ Additionally, the broken crosslinking points caused by stretching during the cycle test did not have enough time to rebuild to the original state, making the hysteresis loop area in the second cycle significantly smaller than that in the first cycle. After resting for 30 min, the loading/unloading curve of the sample was close to the original curve (Figure S7a, Supporting Information), indicating that MGD had good anti‐fatigue performance. Furthermore, after a stretch‐recovery cycle and standing for 30 min, the hysteresis area of the sample was comparable to the initial level (Figure S7b, Supporting Information), suggesting the excellent mechanical recovery ability of MGD.

In the process of recovery after GBR surgery, it is necessary for wound closure materials to have good self‐healing ability to response to the damage caused by daily chewing. As shown in Figure 1e, the artificial scar made on the MGD surface almost disappeared within 2 h at 60 °C, suggesting its good ability to rebuild after surface damage. Thereafter, two separate pieces were joined together at room temperature. Notably, the MGD membrane could still withstand large tensile deformation after self‐healing (Figure 1f). Meanwhile, a similar property was observed in underwater condition (Video S1, Supporting Information), which offers the possibility of its use in soft tissue wound closure, especially in the moist environment of oral cavity. Subsequently, the mechanical properties of MGD after self‐healing were investigated (Figure 1g). It can be seen that with the increase of heating temperature, the ultimate tensile strength and elongation at break of MGD both enhanced, and reached 95% of the original value after healing at 60 °C for 6 h (Figure S8, Supporting Information). All these results demonstrated that MGD has favorable self‐healing ability, and thus providing great prospects as a stable dressing material in practical applications.

### 2.2 Biocompatibility and Antibacterial Activity of MGD

**Figure 2:**
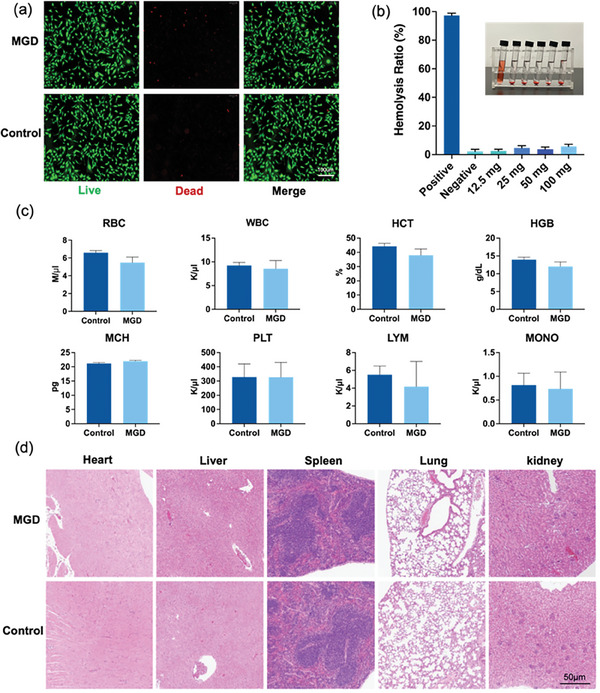
Biocompatibility of MGD in Vitro and In Vivo. a) Live/dead cell staining of MGD group and the control group co‐cultured with oral epithelial cells for 48 h. Scale bar: 100 µm. b) Hemolysis evaluation after RBCs had been incubated with different amounts of MGD for 2 h, using deionized water and PBS buffer as positive and negative controls, respectively. (n = 4). c) MGD does not affect the hematopoietic system of rabbits. The MGD samples (15 × 10 × 0.5 mm) were attached to the wound for 7 days, and their blood examination indicators were compared with that of untreated ones. (n = 3). d) H&E staining of the major organs of rabbits treated with MGD on 7^th^ day. Scale bar: 50 µm.

Since GBR dressing require direct contact with tissues, the biosafety of the MGD is paramount. To comprehensively evaluate the biocompatibility of MGD, tests were performed in vitro (cytocompatibility and hemocompatibility) and in vivo (organs toxicity). The results of cell viability assay showed that there was no significant differences between different concentration of MGD and control groups after co‐culture with human oral epithelium cells for 48 h (Figure S9, Supporting Information). This indicates that MGD exhibits excellent biocompatibility with key healing cells during the wound closure process. The live/dead staining showed almost all cells had a strong green signal and normal spindle morphology (**Figure** **2**a), indicating that the MGD had no adverse effects on long‐term cell growth in practical applications. A hemocompatibility test was performed by incubating MGD elastomers with phosphate‐buffered saline (PBS) containing red blood cells (RBCs). It can be clearly seen from the results that, in contrast to the bright red of positive control group, all the MGD groups and the negative control group showed a distinct light red (Figure 2b). The hemolysis of all polymer groups <5% (for 100 mg MGD, the ratio was 4.92%), affirming the remarkable blood compatibility of MGD, these results signify a step forward in the potential use of MGD in clinical scenarios.

For in vivo biocompatibility testing, the MGD samples (15 × 10 × 0.5 mm) were attached on rabbits’ oral wound for 7 days (Figure S10, Supporting Information). Thereafter the hematology parameters in serum were studied and compared, including red blood cell (RBC), white blood cell (WBC), hematocrit (HCT), hemoglobin (HGB), platelet (PLT), lymphocytes (LYM), and other blood biochemical parameters. Figure 2c showed no significant difference between the control and MGD groups among different blood examination indicators, suggesting MGD does not affect the hematopoietic systems of rabbits, which is consistent with previous researches.^[^ ^27^, ^28^ ^]^ In addition, hematoxylin and eosin (H&E) staining demonstrated the absence of adverse pathological lesions in major organs (heart, liver, spleen, lung, and kidney) (Figure 2d). These compelling findings not only underscore the biocompatibility of MGD, but also shed light on its promising role in advancing practical applications, particularly in the field of regenerative medicine.

### 2.3 Antibacterial Activity of MGD In Vitro and In Vivo

**Figure 3:**
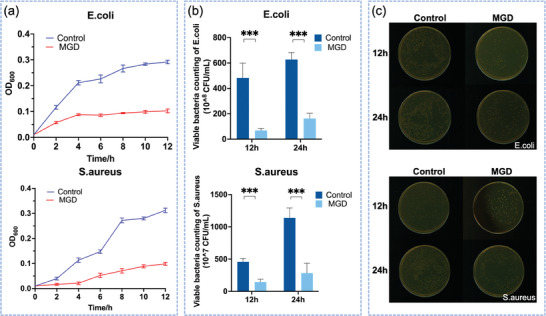
a) OD600 value of bacterial medium of the two common oral bacteria (E.coli and S.aureus) cultured on the MGD and control dressing, (timepoint = 2,4,6,8,10,12 h, n = 3). b) Visible quantity of bacterial colonies of MGD and control group after 12 and 24 hours’ cultivation of E.coli and S.aureus (n = 3), “***” means P<0.001. c) Macroscopic view of bacteria within the culture medium at 12 and 24 hours’ timepoint.

Fortified with CTAB, MGD has good antibacterial properties in the wound healing process of GBR. CTAB is a quaternary ammonium compound with positive charge, while bacteria have a negative charge, so it can bind to negatively charged bacteria through electrostatic action, thereby destroying the bacterial membrane integrity and eventually leading to cell lysis and death.^[^ ^29^, ^30^ ^]^ **Figure** **3**a depicted that in terms of the growth rate of the two commonly encountered oral bacteria (E.coli and S.aureus), the MDG group exhibited a significantly lower rate compared to the control group at 2,4,6,8,10,12 h. The OD_600_ value of the MGD group, at the 12‐h mark, was still below 0.1, while the control group reached beyond 0.3. After 12 and 24 h of cultivation, the visible quantity of bacterial colonies in the control group was noticeably higher than that in the MGD group, which was shown in Figure 3b. The macroscopic view of bacteria within the culture medium was shown in Figure 3c, consistent with the colonies counting results. These findings strongly suggest that the antimicrobial properties of MGD may have a crucial role in sustaining a favorable microbial environment for the healing process, which is attributed to the inclusion of the antimicrobial component CTAB and the outstanding self‐cleaning capability of the MGD.

**Figure 4:**
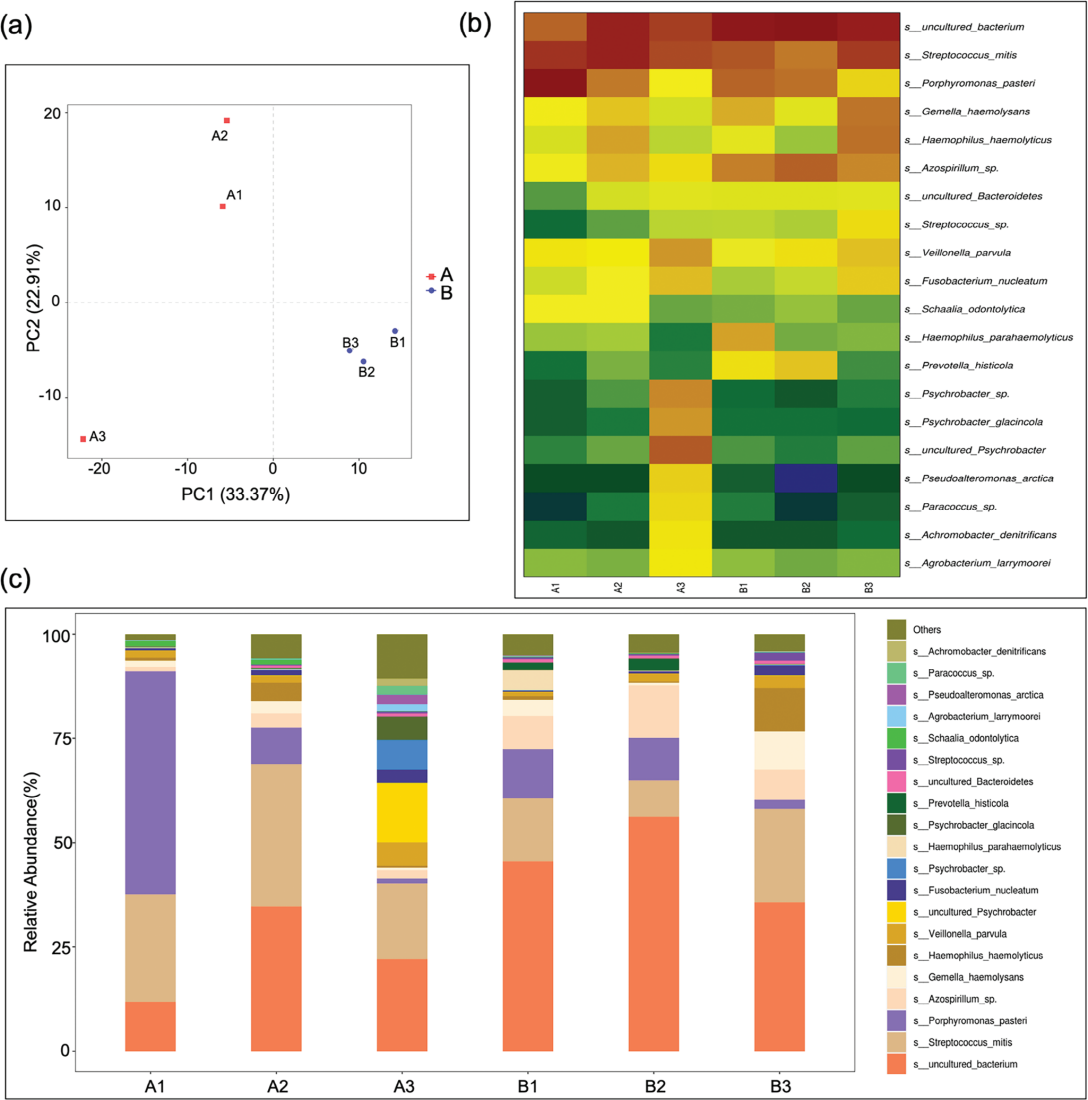
a) MiseqPE300 sequencing and analysis of the 16SV3‐V4 region of wound bacteria samples of the control group (A1, A2, A3) and the MGD group (B1, B2, B3) after 7 days of wound healing (n = 3). b) The OTU and its taxonomic level heatmap of the top‐ranked dominant species of the control group (A1, A2, A3) and the MGD group (B1, B2, B3), and the color depth represents the level of OTU/species abundance. c) Histogram of species composition analysis of the top 20 species in relative abundance of the control group and the MGD group.

In vivo test, after 7 days of wound healing, we took samples from the soft tissue surface of the control group (A1, A2, A3) and the MGD group (B1, B2, B3) and carried out microbial culture. Subsequently, high‐throughput analysis of the 16S V3‐V4 region of the cultured bacteria in groups A and B using the MiSeqPE300 sequencing platform was conducted (gene amplification curve and melt curve are depicted in Figures S11 and S12, Supporting Information). **Figure** **4**a illustrated that the flora diversity in the MGD group is relatively consistent. Simultaneously, it exhibits significant differences compared to the control group. Notably, there are also variations among the three samples within the control group. Quantitative differences among individual samples are presented in Figure S13 (Supporting Information). This suggests the possibility of two distinct wound healing modes between the MGD group and the control group. To delve deeper into this, we conducted an analysis of Operational Taxonomic Units (OTU) and their level heatmap. The results are presented in Figure 4b at the species level. From these results, it is apparent that the control group is significantly influenced by oral environmental bacteria, while the MGD group exhibits a scenario resembling sterile wound healing. Additionally, we generated a sample clustering bar chart, and the results are displayed in Figure S14 (Supporting Information).

Furthermore, the species composition analysis results (Figure 4c) depict the microbiota present in each sample, including the relative abundance of each microorganism. As expected, the majority of bacteria in the control group consist of common oral species, such as Porphyromonas and Streptococcus. Notably, sample A3 also exhibited the presence of a suppurative infection flora Haemophilus and Veillonella. However, to our pleasant surprise, we observed a significant increase in the proportion of uncultured species in the MGD group, along with a marked decrease in the abundance of species causing oral wound infections. This highlights the exceptional antibacterial properties of MGD. Additionally, credit is due to MGD's outstanding self‐cleaning characteristics, vividly demonstrated in Video S2 (Supporting Information). These in‐depth analysis shed light on the underlying mechanisms of MGD's role in oral wound healing. We observed that the microbial composition of the MGD group was closely aligned with aseptic wound healing, whereas the control group exhibited typical oral infectious flora, with the highest abundance consisting of two commonly found pathogenic bacteria, Porphyromonas and Streptococcus, which were also detected in peri‐implant saliva and suggest a high potential for peri‐implant bone loss.^[^ ^31^ ^]^ Comparing our findings with previous research underscores MGD's distinct capacity to inhibit the growth of pathogenic microbes within the oral microbiome, potentially reducing the risk of infections.^[^ ^32^, ^33^ ^]^ Moreover, the analysis of the 16SV3‐V4 region revealed a spectrum of beneficial microbial populations in the MGD group, such as Gemella haemolysans and Azospirillum.^[^ ^34^ ^]^ The disparity in microbial composition may explain the observed lower bacterial growth rate and smoother healing process in the MGD group. The LDA Effect Size analysis further confirms significant differences in species abundance between the two groups, consistent with the species composition analysis, as shown in Figure S15 (Supporting Information). Last, an interrelation analysis was conducted on the top thirty absolute abundance results across all samples using the Spearman correlation test. However, no significant findings were observed (Figure S16, Supporting Information). These findings emphasize the practical implications of our research in improving clinical outcomes and reducing the risk of infections, which is of paramount importance in oral healthcare.

### 2.4 Animal Model and GBR Procedure

**Figure 5:**
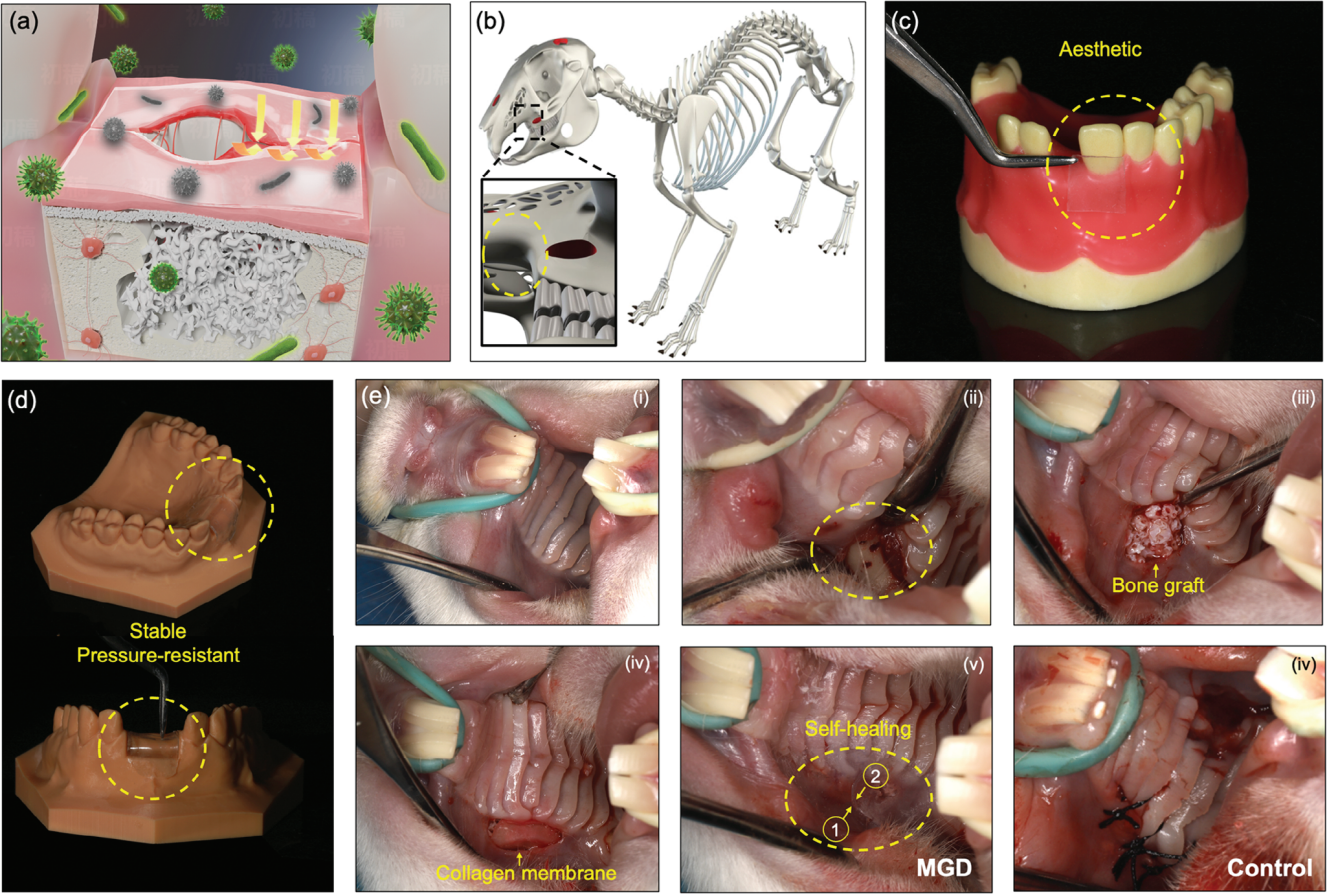
a) Diagram illustrating the functional role of MGD in the oral cavity. b) Experimental use of the New Zealand rabbit model, with the naturally occurring bone defect area in the anterior maxillary molar region serving as an excellent site for GBR surgery. c) MGD's aesthetic characteristics. d) MGD's stable and pressure‐resistant characteristics. e) Intraoral GBR procedure, performed by a specialist with over ten years of clinical implantation experience.

To validate the practical effectiveness of MGD within the oral cavity (**Figure** **5**a), we chose to utilize a rabbit oral model which differs from the conventional use of cranial and tibial models.^[^ ^35^, ^36^, ^37^ ^]^ This choice allowed us to closely mimic human oral biological processes and align with real‐world clinical application scenarios (Figure 5b). The utilization of MGD for wound closure offers the distinct advantage of transparency, which carries significant aesthetic value, particularly in the anterior dental region (Figure 5c). Furthermore, MGD demonstrates robust space maintenance capabilities at bone defect sites, a crucial factor for wound closure during the healing process and subsequent bone formation (Figure 5d).

The surgical procedure diagram is presented in Figure 5e, and the entire surgery was performed by a specialized oral implant dentist with over ten years of clinical experience. The surgical aseptic principles were strictly followed throughout the operation. The approval number for the animal experiment plan of the project is 2022JS‐012, as authorized by the Stomatology Hospital Fudan University Experimental Animal Science Department Animal Welfare and Ethics Committee. A 3‐month‐old male New Zealand rabbit was fixed in supine position and the mouth was fixed open (Figure 5e,i). A 10 mm longitudinal flap incision was made in the maxillary mucosa, and the periosteum was separated. A 0.3mm‐diameter split drill was used to prepare nutrient holes on the bone surface (Figure 5e,ii). The bone xenograft (small granular cancellous bone, Bio‐Oss, Geistlich, Switzerland) (Figure S17, Supporting Information) was implanted above the nutrient holes (bone graft volume of 3 mm height, 4 mm mesial‐distal length, and 3 mm width) (Figure 5e,iii), covered with Megreen oral absorbable bio‐membrane (1.5*2 mm, Shaanxi Reshine Biotech Company, China) (Figure 5e,iv). In the experimental group, MGD was applied to cover and close the wound, through self‐healing properties to transfer mucosal tension to the dressing (Figure 5e,v). Meantime, mucosal flaps were sutured in the control group (Figure 5e,vi). Thanks to the outstanding aesthetic properties of MGD, unless carefully observed, it can be challenging to discern the two layers of self‐healing MGD membranes in (v). This significantly expands its potential applications in the aesthetic zone of the anterior teeth.

### 2.5 MGD Promoted Soft Tissue Healing

**Figure 6:**
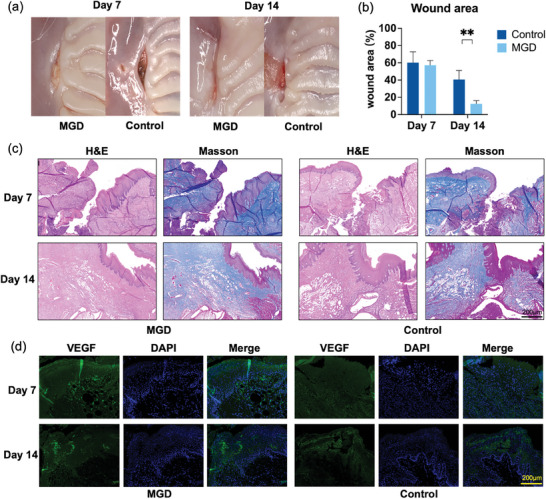
Assessment of soft tissue healing effects of MGD in vivo. a) Soft tissue healing outcome diagrams for the MGD group and the control group on the 7^th^ and 14^th^ day. b) Wound area of soft tissue of MGD group and the control group on the 7^th^ and 14^th^ day, “**” means P<0.01. c) Soft tissues with H&E staining and Masson's trichrome staining of wound sites in the MGD group and the control group on the 7^th^ and 14^th^ day. Scale bar: 200 µm. d) Soft tissues with immunohistochemical fluorescence staining of VEGF marker of the MGD group and the control group on the 7^th^ and 14^th^ day. The blue image indicates cell nuclei labeled with DAPI, while “Merged” represents the fusion of the two former components. Scale bar: 200 µm.

As shown in **Figure** **6**a, on the 7^th^ day post GBR procedure, both the MGD group and the control group were in the process of wound healing. In the MGD group, the wound appeared clean with no food residue, and the collagen membrane was observed to be still intact in its original position. In contrast, the control group exhibited a significant accumulation of residue in the wound area, along with slight swelling of the surrounding soft tissue. Similarly, from the video and figure, it was evident that MGD possesses a strong self‐cleaning and hydrophobic property, making the food residues difficult to adhere to the dressing (Video S2 and Figure S18, Supporting Information). In contrast, sutures accumulated food residues easily. On the 14^th^ day, the wound of MGD group had healed without obvious infection or tear. The wound of control group was still healing, and the speed was significantly slower than that of the MGD group. To quantify the wound healing rate, we employed Image Pro Plus 6.0 (MEDIA CYBERNETICS, USA) to compare the wound area of the two groups on the 7^th^ and 14^th^ day. The calculation method for wound area is presented in Figure S19 (Supporting Information). It was observed that the MGD group exhibited a significantly faster wound healing rate than the control group, especially on the 14^th^ day post‐surgery (Figure 6b).

Masson and H&E staining were employed to examine the microscopic processes involved in soft tissue healing (Figure 6c; Figure S20, Supporting Information). Notably, the MGD group exhibited significantly superior soft tissue healing on both the 7^th^ and 14^th^ day. In contrast, the control group showed evident tissue tearing and poorer healing on the 7^th^ day, accompanied by a notable presence of inflammatory substances in the Masson's staining. By the 14^th^ day, although healing had occurred in the control group, the soft tissue thickness was significantly reduced compared to the MGD group. On the contrary, in the MGD group, the healed soft tissue closely resembled the morphology of the surrounding original tissue. Immunohistochemistry was utilized to mark blood vessels with vascular endothelial growth factor (VEGF), allowing us to observe early vascular growth (Figure 6d; Figure S21, Supporting Information). Angiogenesis is a crucial process in soft tissue healing. In the MGD group, pronounced VEGF expression was observed on the 7^th^ day, indicating active vascularization, which persisted on the 14^th^ day. However, in the control group, this process was less pronounced. The positive fluorescent areas were quantified using Image‐Pro Plus 6.0, and the statistical results are presented in Figures S22 and S23, Supporting Information. These findings not only highlight the beneficial effects of MGD's superior sealing and stabilizing properties on soft tissue healing and angiogenesis. The enhanced vascular stimulation observed in the MGD group carries significant implications. This effect may stem from the improved alignment of wounds, leading to a stable and sterile healing environment. This optimized setting facilitates the smooth transportation of nutrients and oxygen, resulting in accelerated and improved soft tissue healing. Additionally, the robust expression of VEGF could potentially play a crucial role in orchestrating angiogenesis,^[^ ^38^ ^]^ further enhancing blood vessel regeneration within the treated area. In contrast, the slower healing of soft tissue in the control group might be attributed to the tension caused by high suture tension, which hampers wound alignment within an unfavorable oral environment. This notion finds support in pivotal studies such as Smith et al. highlighting angiogenesis's role in accelerating wound healing, by promoting granulation tissue and collagen deposition.^[^ ^39^ ^]^ Further reinforcing this, another research demonstrated that robust neovascularization not only enhances osteogenesis but also expedites the recruitment of osteoprogenitor cells, thus speeding up the process of bone formation.^[^ ^40^ ^]^

Of great significance, the effective closure of initial wounds profoundly impacts GBR, providing a solid foundation for subsequent bone tissue development. Consequently, the exceptional aseptic stable wound healing environment provided by MGD, which induces vascular growth, likely plays a crucial role in the GBR process.

### 2.6 MGD Promoted Bio‐Oss Stability and Early‐Stage Osteogenesis

**Figure 7:**
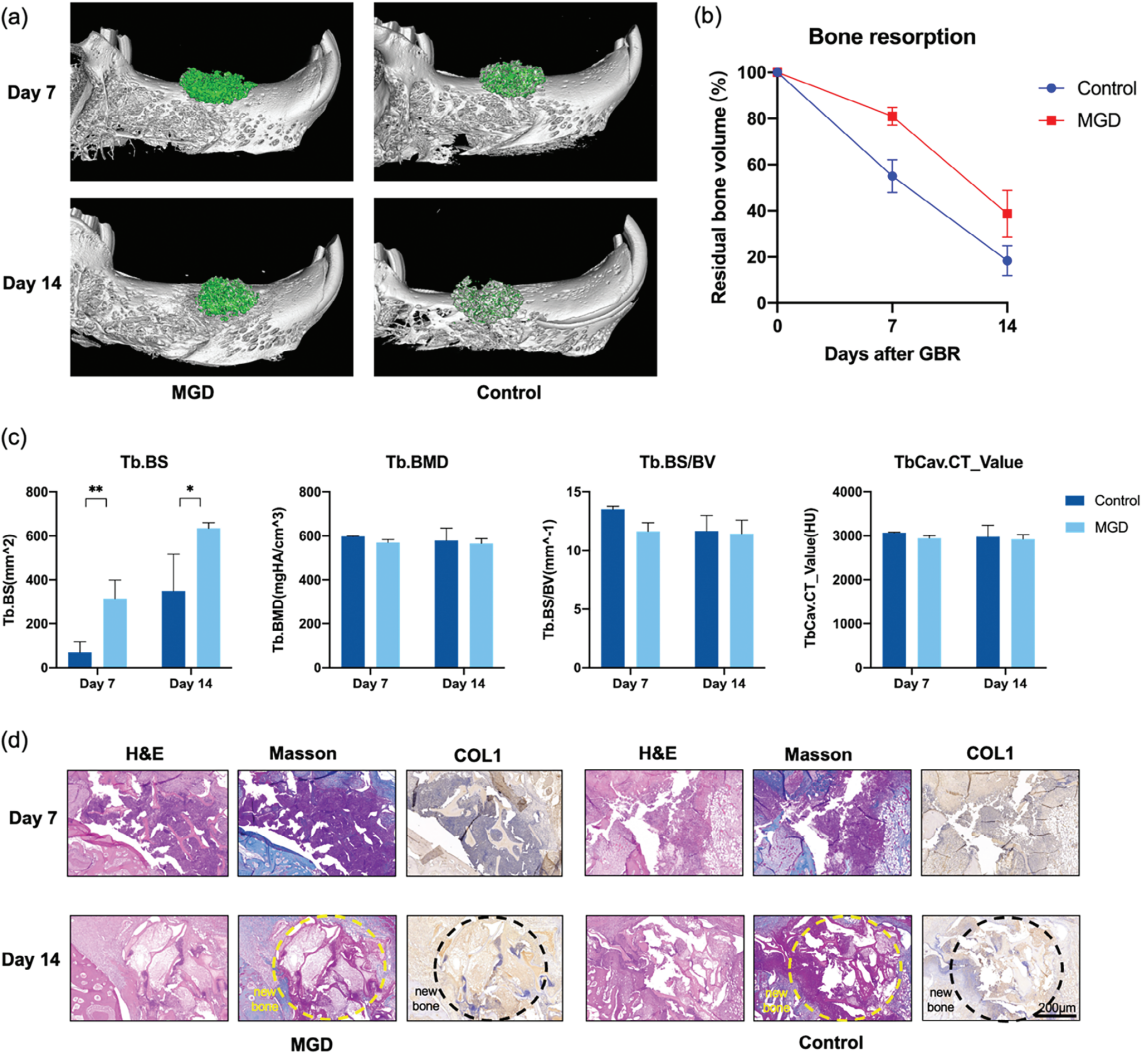
Assessment of effects Bio‐Oss stability and early‐stage osteogenesis of MGD in vivo. a) 3D structure of Bio‐Oss in the MGD group and control group scanned by micro‐CT. The density level is represented by the color green. b) The bone resorption rate determined by comparing the remaining amount of Bio‐Oss to the initial amount, as calculated from the micro‐CT scanning results. c) Several key bone tissue analysis parameters during the process of osteogenesis by MGD and control group of different days. d) Bone tissues with H&E staining, Masson's trichrome staining and immunohistochemical staining of COL1 marker of MGD and control group on the 7^th^ and 14^th^ day. Deeper shades of yellow indicate higher levels of COL1 expression in that region. Scale bar: 200 µm.

The spatial stability of Bio‐Oss during GBR is the key to successful bone regeneration, and the stable three‐dimensional (3D) structure is key to the early infiltration of blood vessels and early osteogenesis. Micro‐CT results showed that the 3D structure of Bio‐Oss in the MGD group was more complete and denser. On the contrary, it could be observed that on the 7^th^ and 14^th^ day, the bio‐oss particles in the control group were looser and the volume was smaller compared to the MGD group. Particularly, on the 14^th^ day, the Bio‐Oss particles in the control group appeared notably dis‐organized and, even worse, had deviated from their originally implanted positions (**Figure** **7**a). Compared with the original implanted bone volume, the resorption rate in the MGD group was significantly lower than that in the control group on the 7^th^ and 14^th^ day (Figure 7b), the detection video for the separated bone graft was presented in Video S3 (Supporting Information). Micro‐CT serves as a tool to evaluate the stability of the experimental group, offering a more comprehensive perspective compared to traditional X‐ray radiographs.^[^ ^41^ ^]^ The clear retention of bone graft material in the MGD group aligns with the concept of stability as a paramount factor in bone graft integration. The analysis of bone parameters in Figure 7c revealed that the bone surface area of the MGD group was significantly greater than that of the control group on both the 7^th^ and 14^th^ day, indicating denser Bio‐Oss in the MGD group per unit volume. Although the bone graft material undergoes continuous absorption, the increased bone contact rate per unit area in the MGD group suggests superior early‐stage bone formation.

H&E and Masson's staining showed that on the 7^th^ day, the Bio‐oss in the MGD group was denser, effectively maintained its original position and established thorough contact with the surrounding blood tissue. In contrast, the control group's bone graft particles had lost significantly, loosely arranged, and exhibited substantial gaps in the surrounding area. By the 14^th^ day, early bone formation was evident in the MGD group, where immature bone trabeculae were visible within the bone graft particles. In contrast, the control group's Bio‐Oss particles still exhibited numerous gaps, and the newly formed tissue remains non‐mineralized. These observations collectively indicated a notably faster bone formation process in the MGD group compared to the control group.

Collagen type I (COL1) served as a pivotal marker of bone formation, as it demonstrated the presence of collagen precursors in bone formation. It could be observed that in the MGD group on the 14^th^ day, there was distinct and strong COL1 positive expression within the bone interstitium, indicating an active process of osteogenesis in that region. Conversely, such expression was scarce in the control group (Figure 7d; Figure S24, Supporting Information). This observation aligns with prior studies illustrating the correlation between enhanced collagen deposition and early‐stage osteogenesis.^[^ ^42^, ^43^ ^]^ The substantial increase in COL1 positive area supports the conclusion that the MGD group promotes early bone formation. Last, the integral optical density values of COL1 positive areas were calculated using Image‐Pro Plus 6.0, and the statistical results are displayed in Figures S25 and S26 (Supporting Information). In summary, these findings underscore the vital role of MGD in enhancing spatial stability during GBR, leading to improved bone regeneration, early vascularization, and accelerated osteogenesis. The denser Bio‐Oss structure, reduced resorption rate, and enhanced bone surface area in the MGD group demonstrate its potential significance in advancing bone regeneration therapies and its promising clinical applications in the field of regenerative medicine.

## 3 Summary and Conclusions

The MGD membrane developed in this study is used in oral GBR, showing its excellent biocompatibility and antibacterial ability. During surgery, it can help to close wounds more tightly and efficiently. Its stable self‐healing properties in moist environment can effectively prevent wound dehiscence, while effectively resisting food residues. In addition, MGD also has more aesthetic properties than conventional sutures, which is of great significance in aesthetic zone surgery. Most importantly, in postoperative testing, MDG film can effectively help wounds to heal quickly, while reducing infection and cracking. It is superior to the traditional suture method in terms of the ability to maintain the space and the stability of the bone grafting materials, as well as the ability of early wound healing. All of the above are in line with the “PASS” principle, which ensures better bone regeneration after surgery.

## 4 Experimental Section

### Materials and Apparatus

Hydroxyl terminated polybutadiene (HTPB, 2100 g mol⁻^1^) was purchased from Cray Valley. (IPDI, 98%), 2‐Hydroxyethyl disulfide (HEDS), the catalyst dibutyltin dilaurate (DBTDL, 95%) and (CTAB, 99%) were obtained from Sigma‐Aldrich. All chemicals were used as received without further purification. NMR (1H) spectra were recorded on Bruker Avance III 400 NMR spectrometer in deuterated solvent at room temperature. Transmittance spectra were recorded on a SHIMADZU UV‐1800 ultraviolet spectrophotometer. Fourier‐transform infrared spectra were recorded on Bruker (Tensor 27) Fourier Transform InfraRed. Contact angle measurements were carried out with JC2000DM.

### Synthesis of MGD

HTPB (2.1 g, 1 mmol) was first heated at 80 °C under vacuum for 2 h to remove any moisture. Then, HEDS (154 mg, 1 mmol) as chain extender was added to HTPB under N_2_ atmosphere. After these two reagents form homogenous viscous liquid, IPDI (467 mg, 2.1 mmol) and DBTDL (5 mg, ≈1600 ppm) were added dropwise into the vessel and stirred until the magnet cannot rotate. Subsequently, the mixture was put into the oven (80 °C) for 8 h. After that, the mixture was dissolved in chloroform to form a homogeneous solution. Then, MeOH (30 mL) was added for precipitation of the product. White precipitate‐like viscous liquid appeared and the mixture was settled for 30 min. Then, the upper solution was decanted. After that, 15 mL chloroform was added to dissolve the product. The dissolution‐precipitation‐decantation was repeated three times. Thereafter, 2.7 mg CTAB (dissolved in chloroform) was added to above solution, and the final mixed homogeneous solution was poured into a Teflon mold and allowed to slowly evaporate at room temperature overnight. Thereafter, the resulting film was dried in a vacuum oven at 80 °C for 24 h to remove residual solvent, resulting in transparent film of MGD.

### Mechanical and Self‐Healing Characterizations

Mechanical tests were performed using a universal Instron. The samples were cut into a small dumbbell shape with a thick ness of ≈0.45 to 0.55 mm. The stretching rate was 100 mm/min unless stated otherwise.

Scratch self‐healing processes were monitored using an optical microscope (BX51M, Olympus) equipped with a camera (LC20, Olympus).

Complete fracture self‐healing measurements were carried out by cutting the samples in half in air, and then two pieces were manually placed in the corresponding position. During the self‐healing process, no external stress was applied to the interface. For under water experiment, MGD samples were immersed in water and cut in half, and then left to heal under the same condition.

### Cytotoxicity Evaluation

Cell Compatibility Test of MGD in vitro: Human oral epithelium cells purchase from Procell (Wuhan, China) were seeded at a density of 3 × 10^3 cells per well in a 96‐well plate and cultured with different concentrations of the polymer (10, 100, and 500 µg mL−1) in the medium. The optical density (OD) was measured at 450 nm using the CCK‐8 assay at 0 and 48 h time points.

The live/dead cell staining: Cells were seeded at a density of 5 × 10^4 cells per well in a 24‐well dish and cultured in medium containing 100 µg of MGD polymer. After 48 h, the Live/Dead working solution was added and incubated for 30 min. Following a PBS wash, cells were observed under an inverted fluorescence microscope.

### Hemolysis Assays

The hemolytic properties of MGD were tested using rabbit blood (n = 4). Rabbit blood (2 mL) was diluted with PBS buffer and then centrifuged at 10 °C for 10 min at 2000 rpm. The RBCs were separated from the serum. The pellet of RBCs was washed with PBS and then diluted with 10 mL of PBS. Subsequently, 1 mL of the diluted RBC suspension was mixed with 5 mL of PBS containing varying masses of MGD. Diluted RBC suspension (1 mL), deionized water (5 mL), and PBS (5 mL) were used as positive and negative controls, respectively. The obtained mixture was incubated at 37 °C for 2 h and then centrifuged at 3000 rpm for 10 min. The resulting supernatant was transferred to a 96‐well plate, and absorbance at 570 nm was measured using a Biotek ELX800 spectrophotometer.

### Biocompatibility Evaluation In Vivo

At 3 months of age, male New Zealand rabbits underwent an incision in the oral palatal mucosa. The incision site was then covered with MGD elastomer for self‐healing (n = 3). The rabbits were euthanized 7days later. Major organs (heart, liver, spleen, lungs, kidneys) were collected for subsequent analysis. H&E staining was performed to assess the toxicity of MGD. Hematological parameters from serum were concurrently collected for complete blood count analysis (collected using EDTAK2 anticoagulant) and blood biochemistry. The sutured group of rabbits was used as the control group. All relevant animal experiments were conducted following the guidelines of the Institutional Animal Care and Use Committee (IACUC).

### Antibacterial Test

In vitro, the growth kinetics of *Staphylococcus aureus* and *Escherichia coli* was studied to evaluated the antibacterial effect of MGD: A 6 mm diameter antimicrobial dressing was immersed in disc of 10 mL of diluted culture medium and incubated at 37 °C. Then, at set time points (0, 2, 4, 6, 8, 10, and 12 h), 100 µL of the culture medium was removed for the growth testing in a microplate reader to evaluate the bacterial growth kinetics of the corresponding bacteria. A group without antibacterial dressing was used as a control (OD = 600 nm). Then, submerge another 35 mm diameter antibacterial dressing disc in 1 mL of S. aureus/E.coli (10^7–10^8 cfu mL⁻^1^) culture medium and incubated at 37 °C for 24 h. A group without antibacterial dressings was used as a control. Subsequently, 100 µL of the culture was removed and spread on the corresponding agar medium. After overnight incubation, counted the number of colonies on the agar plates.

### Animal Experimental Evaluation

To evaluate the function of the MGD in GBR wound closure and Bio‐oss dimensional stability, animal experiments were carried out. The approval number for the animal experiment plan of the project is 2022JS‐012, as authorized by the Stomatology Hospital Fudan University Experimental Animal Science Department Animal Welfare and Ethics Committee. 3‐month‐old male New Zealand rabbits were fixed in supine position and the mouth was fixed open, after general anesthesia with intravenous injection of 4% pentobarbital sodium. A 10 mm longitudinal flap incision was made in the maxillary mucosa in front of the bilateral maxillary first molars, and the periosteum was separated to fully expose the maxilla. A 0.3mm‐diameter split drill was used to prepare nutrient holes on the bone surface, and a horizontal guided bone augmentation operation was performed. The bone xenograft (small granular cancellous bone, Bio‐Oss, Geistlich, Switzerland) was implanted above the nutrient holes (bone graft volume of 3 mm height, 4 mm mesial‐distal length, and 3 mm width), covered with MoRui absorbable collagen film (1.5*2 mm, Ruisheng Biotechnology Company, China). The buccal and palatal mucosa was fully separate to relieve tension.

In the experimental group, multi‐functional GBR dressing (MGD) was applied to cover the wound. After adhering to the bilateral mucosal flaps, a complete dressing was formed through self‐healing properties while transferring mucosal tension to the dressing. Meantime, 4‐0 non‐absorbable surgical sutures were used to suture the mucosal flaps in the control group. The surgical aseptic principles were strictly followed throughout the operation.

### Micro‐CT Analysis

To detect the effect of MGD and traditional suture of soft tissue on the spatial stability of Bio‐Oss, Micro‐CT of the grafting area was taken immediately after GBR, which is T0, and recorded the initial bone grafting range. On T1 = 7^th^ day and T2 = 14^th^ day of soft tissue healing, Micro‐CT was taken again to record the spatial distribution of Bio‐Oss, and several bone analysis parameters (Tb.BV; Tb.BS; Tb.BS/BV; Tb.BMD; Tb.Th; TbCav.CT_Value) were measured at the same time to judge evaluate the spatial stability of Bio‐Oss and the imaging indicators of early osteogenesis.

### Histological Analysis

The obtained cranial bone specimens were fixed in 4% PFA for 24 h, then washed with water for 6 h, and subsequently decalcified using 10% EDTA (Servicebio, China). Following this, paraffin sections (6 µm thick) were obtained using a Leica microtome. Some sections were stained using H&E and Masson's trichrome staining methods.

Additionally, to assess angiogenesis and osteogenic activity, immunofluorescence staining and immunohistochemistry staining was performed to detect the expression levels of angiogenesis and osteogenesis‐related proteins, VEGF and COL1. In brief, paraffin sections were deparaffinized, rehydrated in graded alcohols, treated with 0.25% Triton X‐100 for 20 min, and then blocked with 5% BSA for 30 min. Subsequently, primary antibodies against VEGF and COL1 were added, followed by overnight incubation. The sections were then washed with tris‐buffered saline containing Tween 20 (TBST), and Alexa‐488 fluorescent secondary antibodies were added for visualization, with nuclear staining performed. Finally, the expression levels of the target proteins were observed under an inverted fluorescence microscope, and fluorescence intensity was analyzed using Image Pro Plus 6.0 (MEDIA CYBERNETICS, USA). For immunohistochemistry, HRP Polymer (secondary antibody) was added by dripping and incubated at room temperature for 30 min. The chromogen (DAB) was allowed to develop for 3–15 min, followed by counterstaining, dehydration, clearing, and mounting.

### Statistical Analysis

Data were presented as mean ± standard deviation (SD). The sample size (n) for each group included in the analyses is specified in the figure legends. Statistical significance was assessed using GraphPad Prism software (version 7.00). When comparing multiple groups, one‐way Analysis of Variance (ANOVA) was conducted, followed by Tukey's post hoc test to correct for multiple comparisons. For comparisons between two groups, Student's t‐test was employed. The significance level was set at p < 0.05. The symbols used to denote levels of significance in the figures are as follows: * for P < 0.05, ** for P < 0.01, and *** for P < 0.001. Assumptions of normality and homogeneity of variances were verified for each statistical test to validate the applicability of the chosen methods.

## Conflict of Interest

The authors declare no conflict of interest.

## Supporting information

Supporting Information

Supporting Information

Supporting Information

Supporting Information

## Data Availability

Research data are not shared.
