# Supplementary material for: Anti‐Wound Dehiscence and Antibacterial Dressing with Highly Efficient Self‐Healing Feature for Guided Bone Regeneration Wound Closure
Source: Adv Healthc Mater. 2024 Mar 8;13(16):2304128. doi: 10.1002/adhm.202304128 (PMC11468911; doi:10.1002/adhm.202304128)
Supplement: Supplementary file 1 — Supporting Information [file ADHM-13-2304128-s001.pdf]

ADVANCED HEALTHCARE MATERIALS

Supporting Information

for *Adv. Healthcare Mater.*, DOI 10.1002/adhm.202304128

Anti-Wound Dehiscence and Antibacterial Dressing with Highly Efficient Self-Healing Feature for Guided Bone Regeneration Wound Closure

Shenghao Xue, Ning Tang, Cheng Zhou, Shuobo Fang, Hossam Haick, Jiao Sun* and Xueying Wu**

Supporting Information

Anti-Wound Dehiscence and Antibacterial Dressing with Highly Efficient Self-Healing Feature for Guided Bone Regeneration Wound Closure

Shenghao Xue, Ning Tang, Cheng Zhou, Shuobo Fang, Hossam Haick, Jiao Sun*, Xueying Wu**

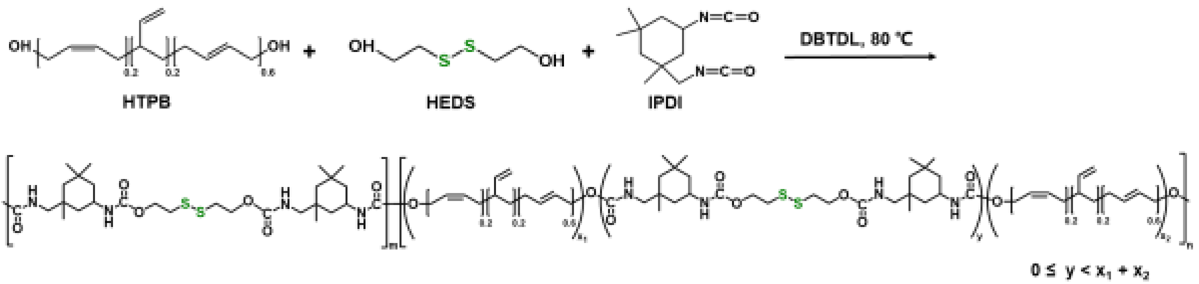

Figure S1. The polymerization reaction and chemical structure of PUIDS.

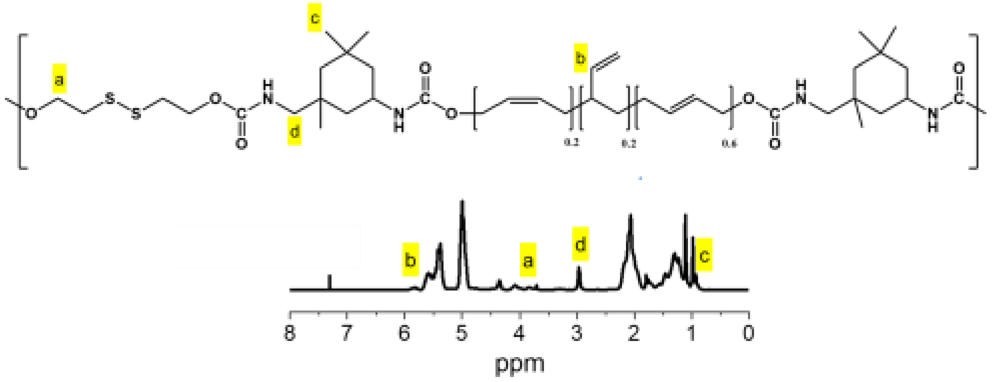

Figure S2. 1H-NMR spectra of different PUIDS

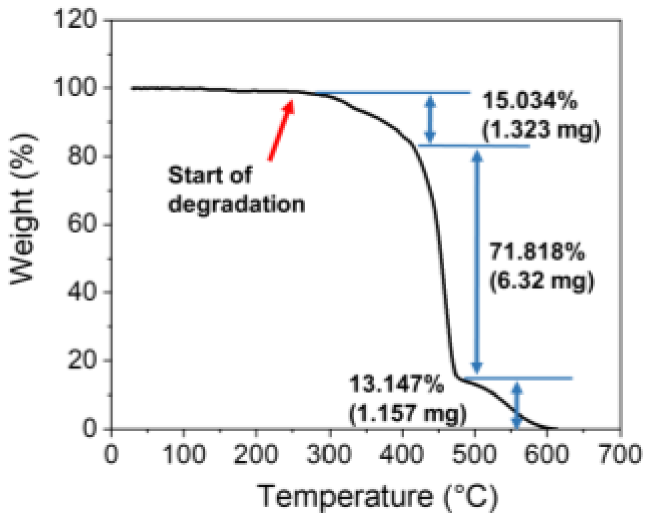

Figure S3. TGA analysis of MGD at 20°C/min under air atmosphere.

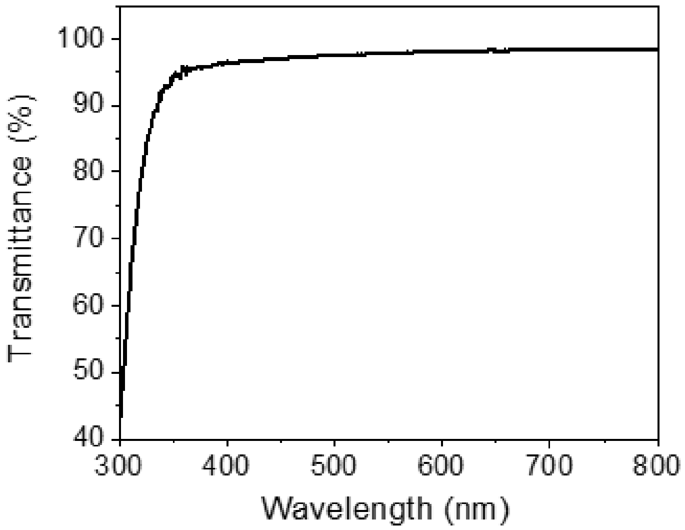

Figure S4. Transmission spectra for MGD films with a thickness of 200 μm.

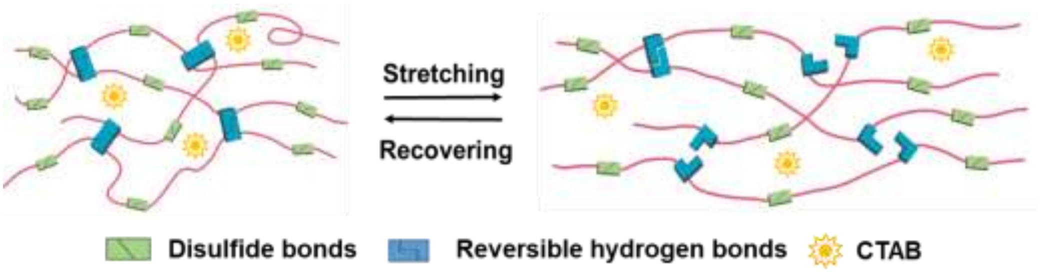

Figure S5. Schematic of the stretching and recovering procedure for MGD.

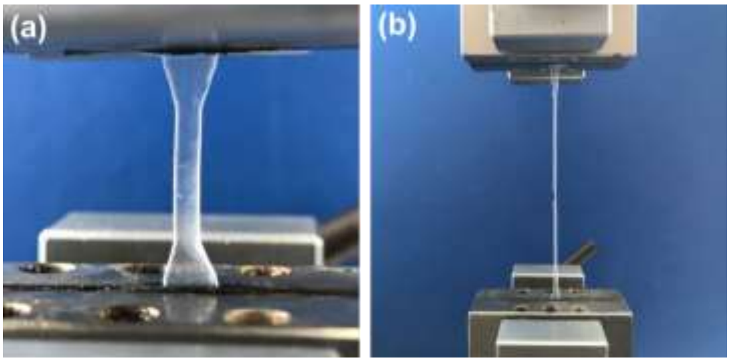

Figure S6. Optical microscopy images of a notched MGD film before stretch (left) and at a certain strain (right).

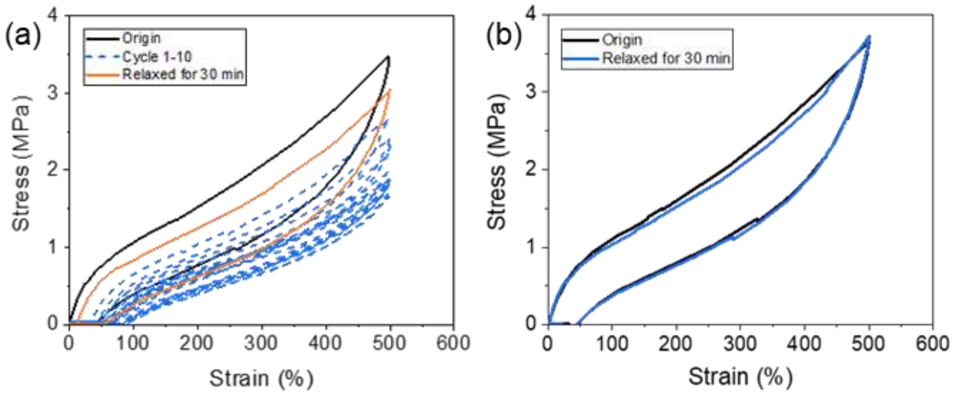

Figure S7. a) Cyclic stress-strain curves of 11 times successive loading/unloading processes of polymer without rest (black and blue), and the 12th loading/unloading cycle after resting at room temperature for 30 min (orange). b) Self-recovery of the loading/unloading curves of MGD.

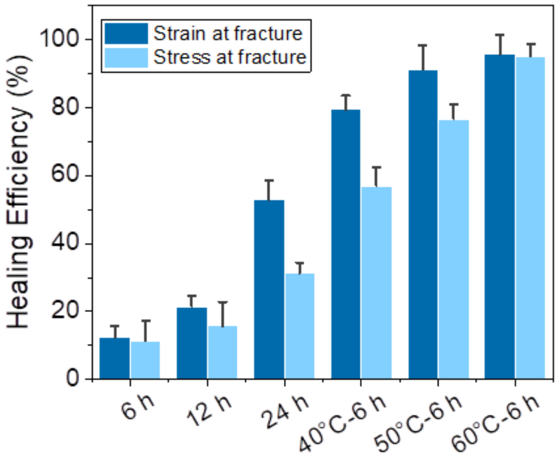

Figure S8. Columns representing the healing efficiencies of polymers under different healing time and conditions.

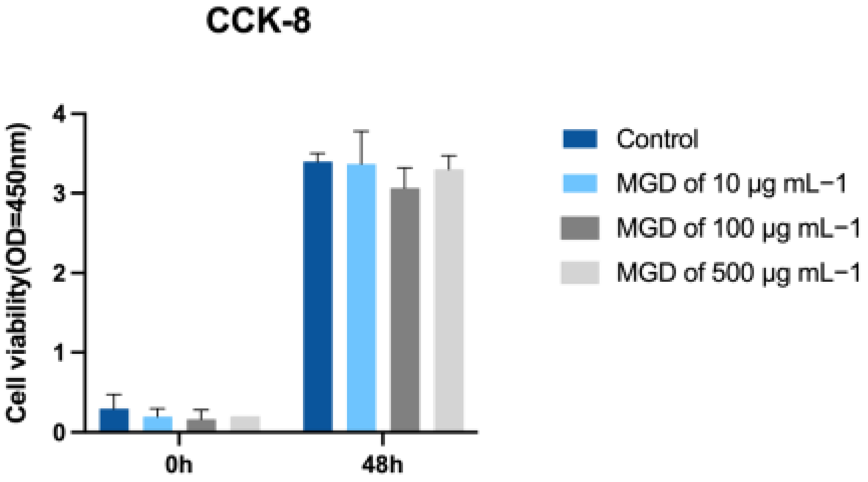

Figure S9. Cell viability of human oral epithelium cells after being co-cultured with different concentration of MGD for 48 h.

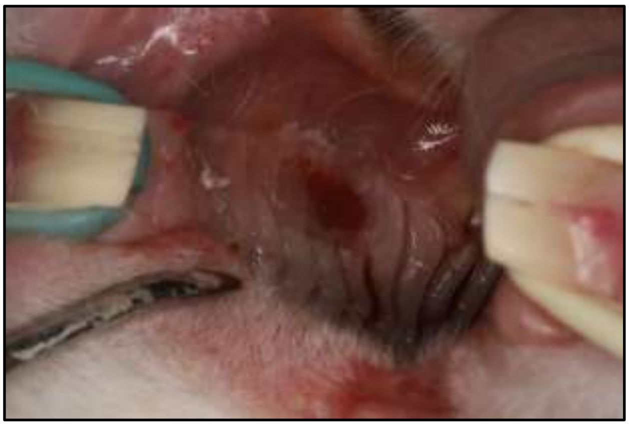

Figure S10. The MGD samples (15 × 10 × 0.5 mm) attached on rabbits' oral wound

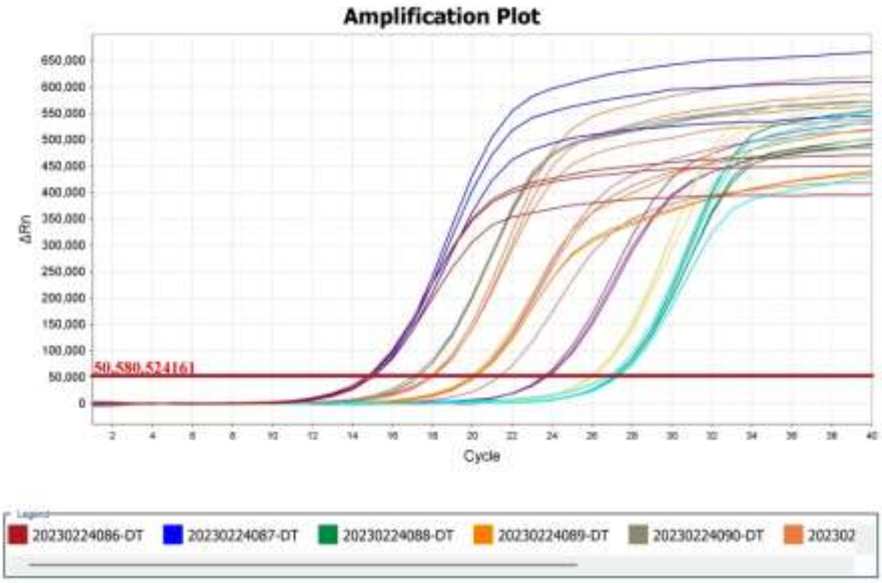

Figure S11. Gene amplification curve of the 16S V3-V4 region.

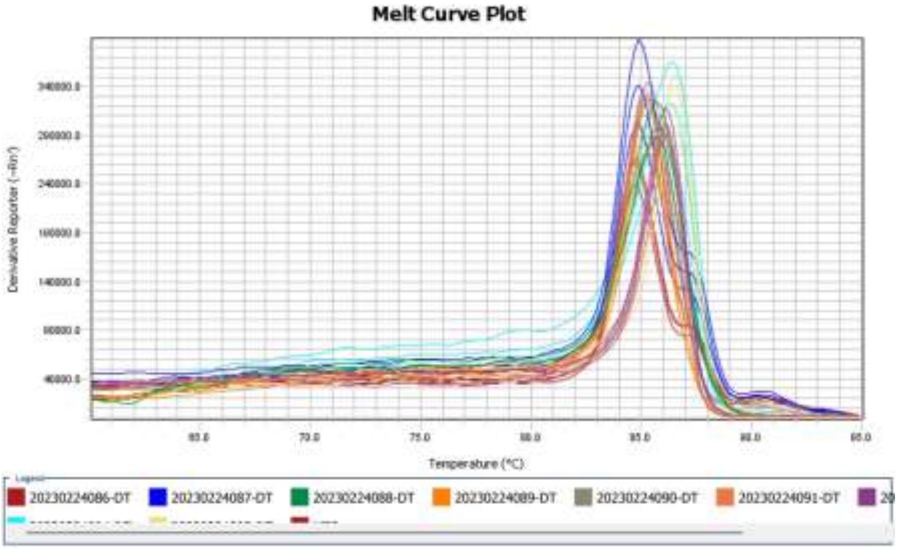

Figure S12. Gene melt curve of the 16S V3-V4 region.

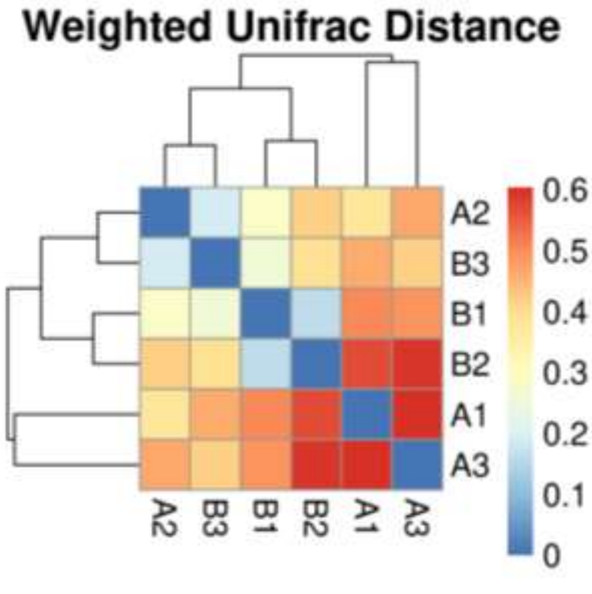

Figure S13. Visualization based on UniFrac heatmap.

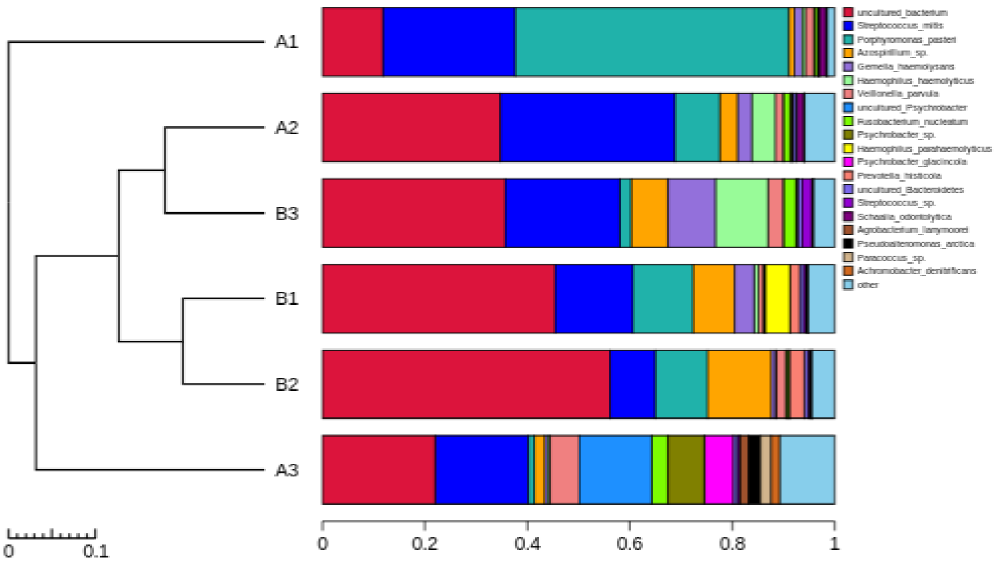

Figure S14. Sample's top 20 species clustering bar plot. The x-axis represents the relative abundance of species within each sample, while the y-axis indicates the sample names.

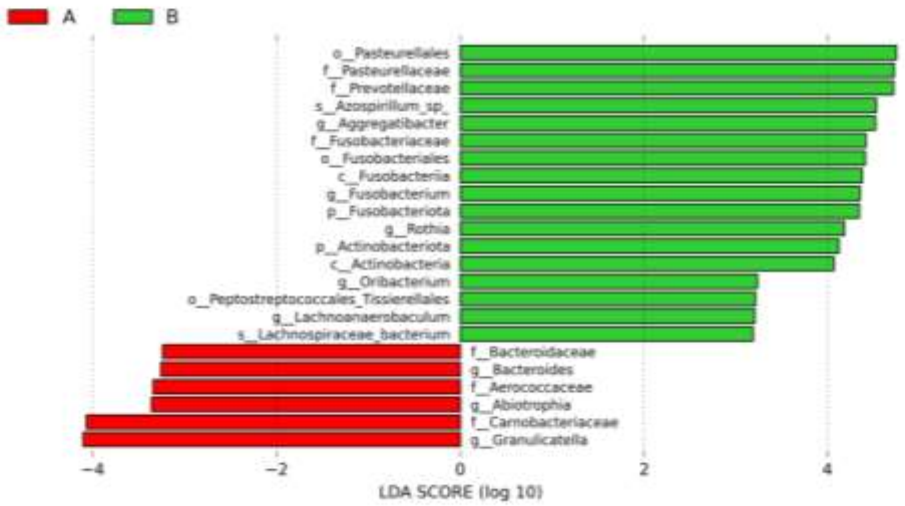

Figure S15. LDA effect size distribution bar plot based on classification-driven LEfSe analysis. The bar chart depicts species exhibiting significant abundance variations across different groups, with the length of each bar representing the magnitude of the impact of these distinct species

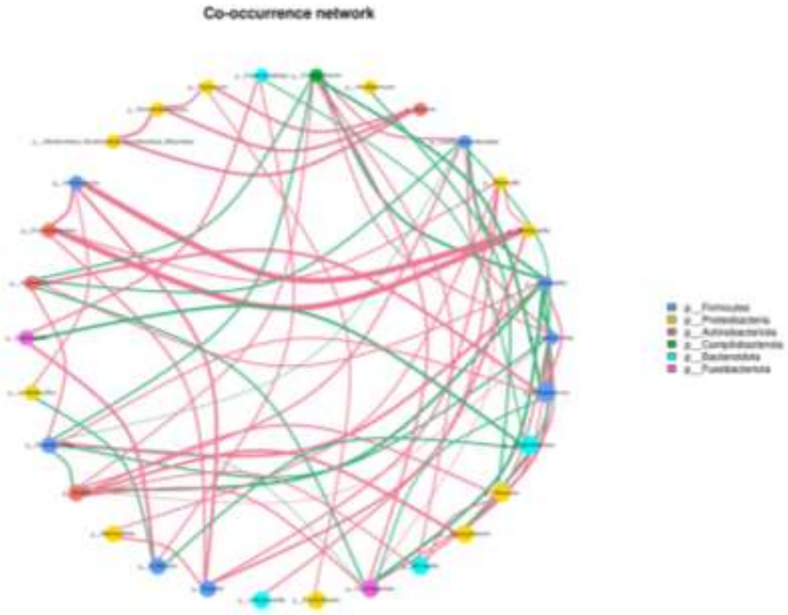

Figure S16. Network Interaction Diagram. The size of the dots represents the abundance magnitude, the thickness of the lines indicates the strength of correlation, and the color of the dots represents their respective phyla. Red lines signify positive correlations, while green lines signify negative correlations.

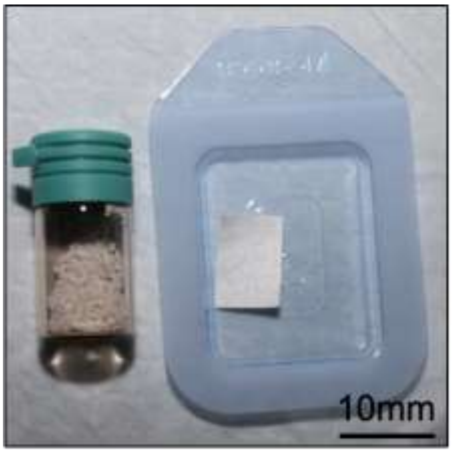

Figure S17. The bone xenograft (small granular cancellous bone, Bio-Oss, Geistlich, Switzerland) and Megreen oral absorbable bio-membrane (1.5*2mm, Shaanxi Reshine Biotech Company, China).

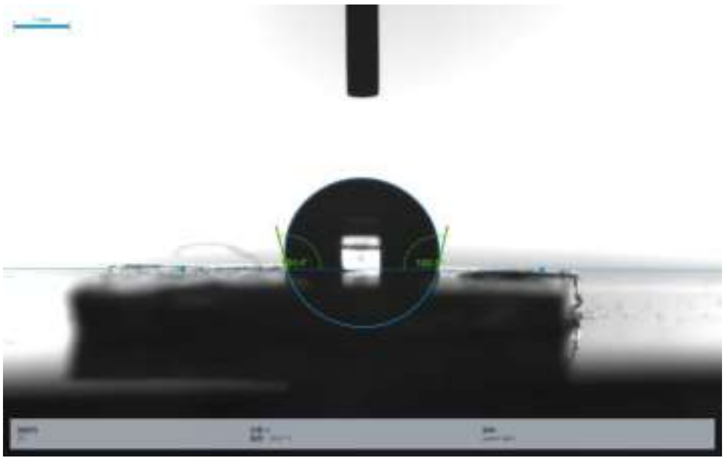

Figure S18. Hydrophobic property test of MGD.

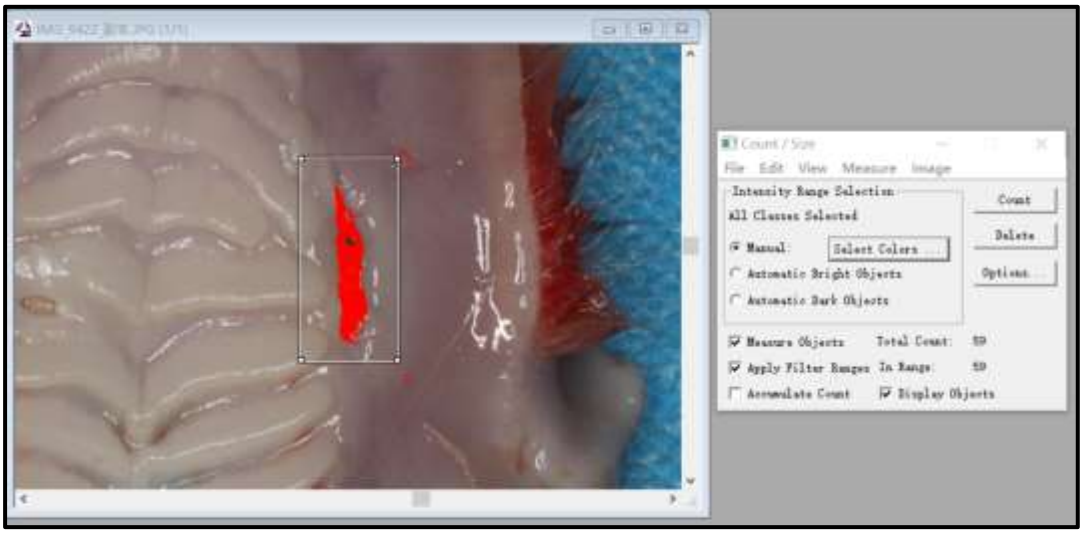

Figure S19. Calculation method for wound area used by Image Pro Plus 6.0 (MEDIA CYBERNETICS, USA).

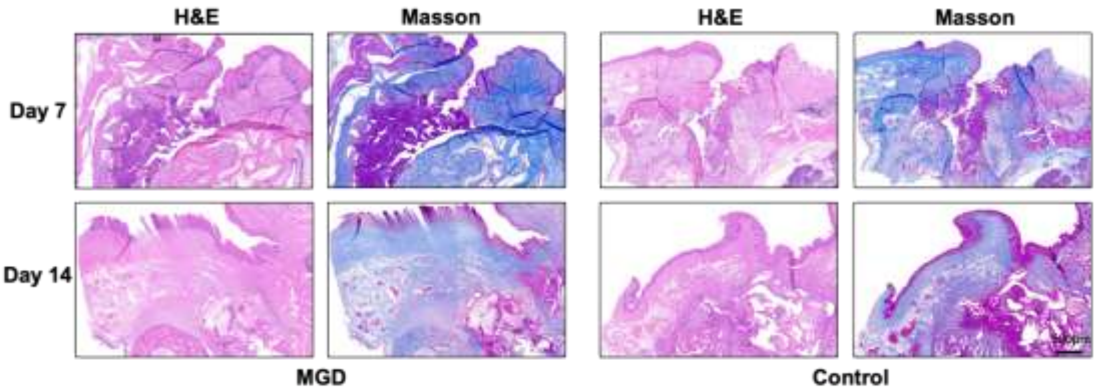

Figure S20. Soft tissues with H&E staining and Masson's trichrome staining of wound sites in the MGD group and the control group on the 7th and 14th day. Scale bar: 500μm.

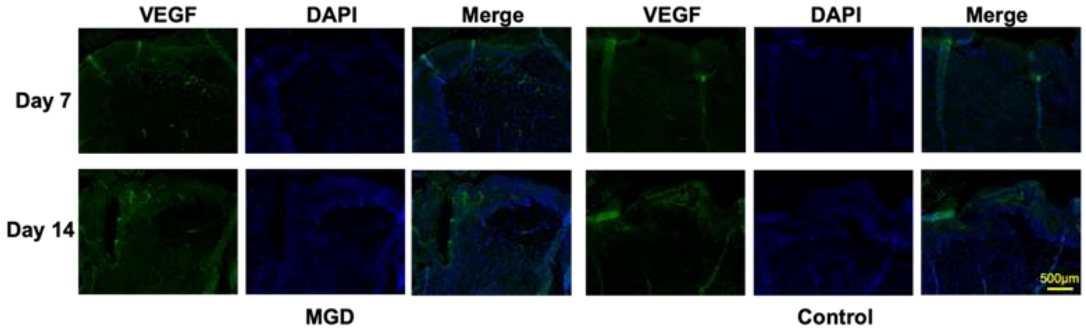

Figure S21. Soft tissues with immunohistochemical fluorescence staining of VEGF marker of the MGD group and the control group on the 7th and 14th day. The blue image indicates cell nuclei labeled with DAPI, while "Merged" represents the fusion of the two former components. Scale bar: 500μm.

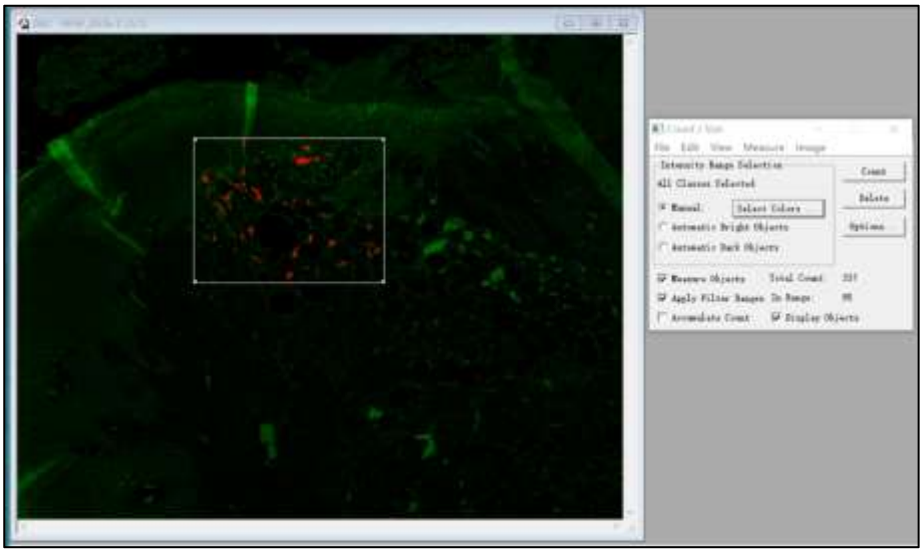

Figure S22. Calculation of the positive fluorescent marked by the VEGF.

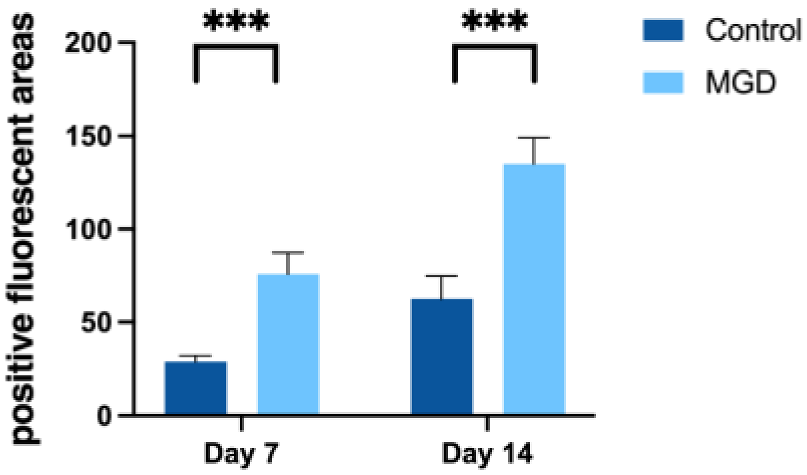

Figure S23. Statistical results of the positive fluorescent areas marked by the VEGF

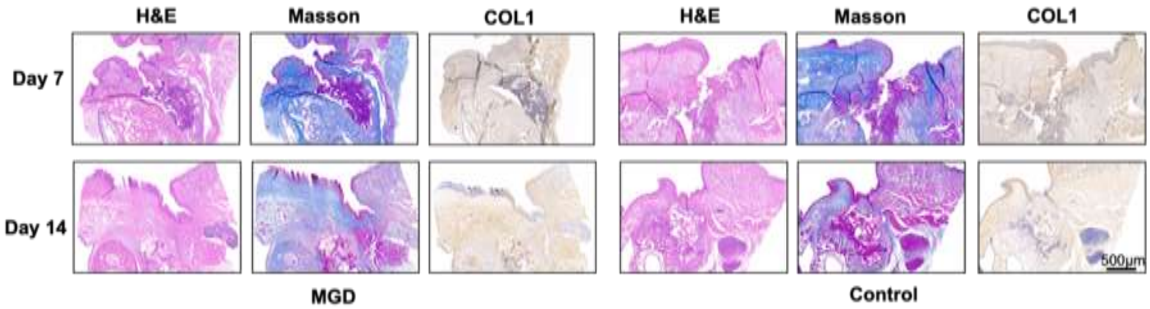

Figure S24. Bone tissues with H&E staining, Masson's trichrome staining and immunohistochemical staining of COL1 marker of MGD and control group on the 7th and 14th day. Deeper shades of yellow indicate higher levels of COL1 expression in that region. Scale bar: 500μm.

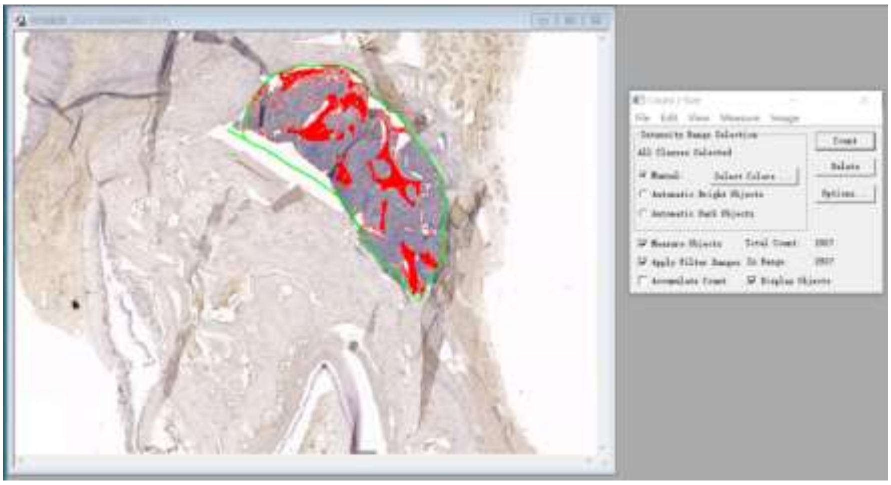

Figure S25. Calculation of the integral optical density values of immunohistochemical positive areas.

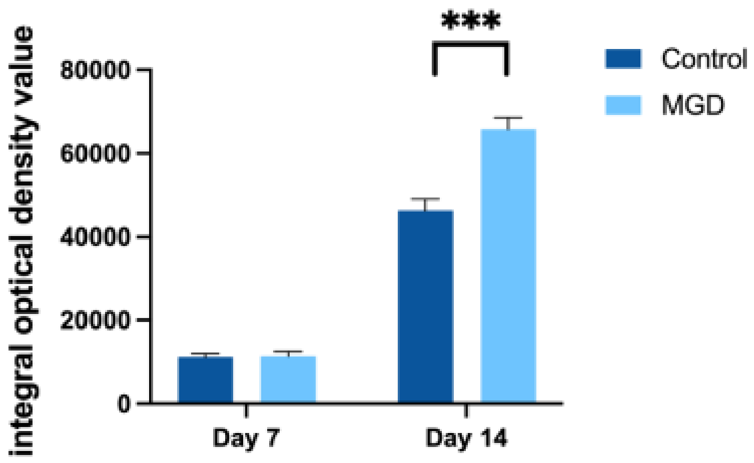

Figure S26. Statistical results of thentegral optical density values of positive immunohistochemical positive areas marked by the COL1.
